# Patterns in Weight and Physical Activity Tracking Data Preceding a Stop in Weight Monitoring: Observational Analysis

**DOI:** 10.2196/15790

**Published:** 2020-03-17

**Authors:** Kerstin Frie, Jamie Hartmann-Boyce, Susan Jebb, Jason Oke, Paul Aveyard

**Affiliations:** 1 Department of Primary Care Health Sciences University of Oxford Oxford United Kingdom

**Keywords:** self-monitoring, self-regulation, weight loss, activity trackers, mobile applications

## Abstract

**Background:**

Self-regulation for weight loss requires regular self-monitoring of weight, but the frequency of weight tracking commonly declines over time.

**Objective:**

This study aimed to investigate whether it is a decline in weight loss or a drop in motivation to lose weight (using physical activity tracking as a proxy) that may be prompting a stop in weight monitoring.

**Methods:**

We analyzed weight and physical activity data from 1605 Withings Health Mate app users, who had set a weight loss goal and stopped tracking their weight for at least six weeks after a minimum of 16 weeks of continuous tracking. Mixed effects models compared weight change, average daily steps, and physical activity tracking frequency between a 4-week period of continuous tracking and a 4-week period preceding the stop in weight tracking. Additional mixed effects models investigated subsequent changes in physical activity data during 4 weeks of the 6-week long stop in weight tracking.

**Results:**

People lost weight during continuous tracking (mean −0.47 kg, SD 1.73) but gained weight preceding the stop in weight tracking (mean 0.25 kg, SD 1.62; difference 0.71 kg; 95% CI 0.60 to 0.81). Average daily steps (beta=−220 daily steps per time period; 95% CI −320 to −120) and physical activity tracking frequency (beta=−3.4 days per time period; 95% CI −3.8 to −3.1) significantly declined from the continuous tracking to the pre-stop period. From pre-stop to post-stop, physical activity tracking frequency further decreased (beta=−6.6 days per time period; 95% CI −7.12 to −6.16), whereas daily step count on the day’s activity was measured increased (beta=110 daily steps per time period; 95% CI 50 to 170).

**Conclusions:**

In the weeks before people stop tracking their weight, their physical activity and physical activity monitoring frequency decline. At the same time, weight increases, suggesting that declining motivation for weight control and difficulties with making use of negative weight feedback might explain why people stop tracking their weight. The increase in daily steps but decrease in physical activity tracking frequency post-stop might result from selective measurement of more active days.

## Introduction

### Background

The repeated measurement and tracking of weight over time, also referred to as self-monitoring, is a common component of weight loss interventions and is consistently associated with greater weight loss [1]. The effectiveness of this self-monitoring strategy is attributed to a self-regulation process [2,3], which posits that people who monitor their weight use the information to reflect on the effectiveness of previous actions and plan further weight loss behaviors, thus engaging in self-experimentation. Furthermore, any discrepancies between desired and actual weight loss progress identified through self-monitoring are hypothesized to trigger corrective action [4].

The frequency of self-weighing seems to play a crucial role, as many studies have revealed significant positive associations between the frequency of self-monitoring and weight outcomes [1,5-7]. One study investigating patterns in self-weighing frequency and body weight measures found that periods of daily self-weighing were associated with weight loss, whereas breaks in weighing were linked to weight gain [8].

Self-monitoring weight has become considerably easier with mobile phone tracking apps and smart scales. These digital tracking devices increase adherence, that is, sticking to a regular frequency of monitoring, and improve weight loss outcomes [9,10], possibly because seeing progress increases motivation and keeps users on track with their goals [11]. However, data suggest that many people do not manage to continue weight tracking long term. In a weight loss trial, the percentage of participants adherent to self-weighing dropped from roughly 70% in the first week to 20% after 70 weeks [12]. Another study found that 25.0% (37/148) of the participants who were asked to weigh daily reduced their monitoring frequency significantly across the study, and approximately 8.8% (13/148) of the participants stopped weighing themselves altogether after 33 weeks [13]. Steinberg et al [14] observed that the average frequency of self-weighing of the participants in the daily weighing intervention group decreased from 6.1 to 4 days per week within 6 months, and 13% (6/47) of the participants stopped weighing themselves completely after the sixth month. Similarly, the frequency of physical activity tracking also declines over time. An observational study showed that, on average, people stop using their physical activity tracking devices after 129 days [15]. In another study, 80% (39/49) of the participants were no longer using their tracking device after 2 months, and 45% (22/49) of the participants did not intend to use it again in the future [16]. Considering that these rates of decline were found in a population of highly motivated individuals (ie, participants in a study), it seems plausible that long-term adherence to tracking may also be low in nonresearch contexts.

Research investigating why people stop using self-monitoring devices suggests that cost, concerns surrounding data sharing, flaws in the design and user experience of technology, as well as issues with data accuracy deter use of self-monitoring technologies [16-20]. Other user-internal reasons for abandonment have also been identified, including users experiencing a mismatch between their expectations and needs and the devices’ capabilities, the users feeling that they have reached data saturation and can no longer learn from their data, and the users having reached their goals [16,20]. However, these data were collected from questionnaires and interviews occurring after abandonment and may suffer from participants retrospectively justifying their abandonment. Here, we use a prospective observational design to examine patterns preceding a stop in self-monitoring using actual tracking data from the Withings Health Mate app (Withings SA, France).

### Objectives

Our first research question (RQ1) considered whether the pattern of weight and physical activity measurements and physical activity tracking frequency changes before people who are trying to lose weight cease weight monitoring. Approximately 80% of people approach weight loss by increasing their physical activity [21-23], so we considered physical activity measurements as a proxy for the engagement in weight loss behaviors and the motivation to lose weight. We tested two competing hypotheses against each other. First, that in the weeks preceding a stop in weight tracking, motivation to lose weight and track behavior would remain high (ie, stable levels of physical activity and physical activity tracking), but the user would receive weight readings that show unsatisfying weight loss progress (such as stable weight or weight gain), leading to frustration and perceived lack of ability to take control of the weight loss progress, making the user stop tracking his or her weight. The alternative hypothesis was that declining motivation for weight loss would manifest in declining weight loss efforts (as measured through decreasing physical activity). This would lead to unsatisfactory feedback, such as weight stability or gain, and to ceasing self-monitoring, potentially because of negative emotions such as shame.

Our second RQ examined physical activity data after users stopped tracking their weight. Our hypothesis was that ceasing self-monitoring of weight would undermine the motivation to engage in weight loss behaviors. We, therefore, expected to see a decrease in physical activity and physical activity tracking frequency after the stop in weight monitoring.

## Methods

### Dataset

The data provided by Withings comprised weight and physical activity records from 438,688 Withings Health Mate app users from January 1, 2014, to January 19, 2018. Withings Health Mate app users consented to the use of their data for research purposes by accepting the terms and conditions of Withings [24].

The dataset consisted of users who (1) were overweight when they started using the app (BMI ≥25 kg/m^2^), (2) had set themselves a weight loss goal in the app, (3) used both the weight and the physical activity tracking features of the app, and (4) synchronized their weight data from Withings smart scales (as previous research indicates that users may underreport unfavorable weight measurements if done manually [25]).

Withings smart scales synchronize weight data to the app via Wi-Fi or Bluetooth. Smart scale owners can set up accounts for up to eight users, and the scale differentiates between users automatically during weighing. Weight measurements are synchronized to the app separately for each user. Physical activity is operationalized as daily step counts in the Health Mate app. The data can be synchronized via Bluetooth from Withings physical activity trackers and Apple watches or via linking to the Google Fit or Samsung Health app. The dataset included demographic information about each user, including gender, age, location, self-reported height, initial BMI, and target BMI.

### Study Design and Data Screening

Only users who stopped tracking their weight for at least six weeks at some point between 2014 and 2018 were included in the analysis. We chose a period of 6 weeks to signify a stop to ensure that the lack of measurements was unlikely to be because of travel. To be eligible, participants also had to have a preceding 16-week phase of consistent weight and physical activity tracking (≥3 measurements per week), to ensure that only users who monitored their weight and physical activity regularly before the stop were included. A case-crossover design [26] was employed for both RQs. For RQ1, 4 weeks of data preceding the minimum 6-week long stop in weight tracking were compared with 4 weeks of data from the same user from the phase of consistent weight and physical activity tracking. We analyzed a 4-week period as we expected that the frequency of weight tracking would change gradually. For the 4 weeks preceding the stop in weight monitoring, there were no minimum requirements for monitoring frequency. Only users starting both analysis time periods with a BMI of 25 kg/m^2^ or above were included. For RQ2, the analysis further included a third 4-week period, taken from the 6-week long break in weight monitoring, that is, post-stop. Where users had several 26-week periods that fulfilled the abovementioned criteria, the first period was analyzed. Previous research has shown that when individuals have set themselves an ambitious goal, they are more likely to be dissatisfied with their weight loss progress [27], probably because these goal weights are harder to achieve. Hence, if people are far away from their weight loss goal and receive frustrating weight measurements, they might also be more likely to disengage from their weight loss attempt. Here, users may have had significant changes in their weight between the two time periods analyzed, thus leading to different distance to goal weight scores. To ensure that these differences and their potential impact do not affect our analyses, we decided to control for the distance to goal weight.

### Analysis

All analyses were conducted in R (version 3.4.1, R Development Core Team 2017, University of Auckland, New Zealand). A statistical analysis plan was published on Open Science Framework preceding the analyses [28].

#### Variables

The independent variables in the analyses were called time period and distance to goal weight. Time period was a factor with 2 or 3 levels (continuous tracking; pre-stop; and, in RQ2, post-stop) and defined which time period the dependent variable in question originated from. The continuous tracking period was located within the 16-week period of consistent tracking during which users recorded at least three weight and physical activity measurements per week. The pre-stop period was located immediately before the stop in weight tracking. The post-stop tracking period (RQ2 only) was located within the 6-week break in weight tracking. Which weeks the time periods encompassed differed slightly by analysis and is described for each analysis separately below. The distance to goal weight variable was calculated as a difference score between the weight measurement closest to the last date of the time period analyzed and goal weight.

To set up the analyses as within subject, we added the variable user ID as a random factor to all analyses. In RQ2 only, we also included the date of measurement as an independent variable to identify when the dependent variable analyzed was measured.

There were three dependent variables. The first was weight change, calculated as the difference between the first and the last measurement of each time period. The second was daily steps. For RQ1, this variable was calculated as the average daily step count across all days of a time period. For RQ2, the individual daily step measurements were used. In both cases, the daily steps variable was divided by 1000 to aid interpretation of the coefficients. The third dependent variable was physical activity tracking frequency, which was calculated as a sum score for the number of days for which physical activity measurements were available in each time period. As physical activity was treated as a proxy measure for motivation to lose weight, increases in daily step counts were interpreted as a strengthened weight loss effort and increased motivation to lose weight. Physical activity tracking frequency was interpreted as motivation to self-monitor weight loss efforts.

#### Research Question 1

**Figure 1:**
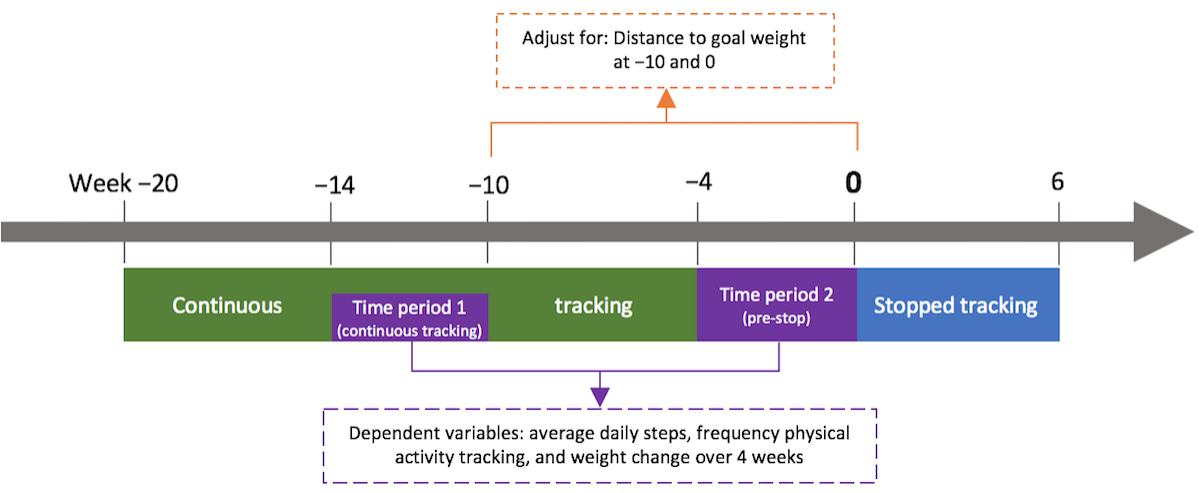
Case-crossover study design to establish linear mixed effects models for research question 1.

For RQ1, we assessed whether average daily steps, weight change, and frequency of physical activity tracking differed between the two time periods: continuous tracking (weeks −14 to −10) and pre-stop (weeks −4 to 0, 29 days each). Figure 1 depicts an overview of the design.

Descriptive analysis examined the demographic characteristics of the analyzed sample and unadjusted differences between the two time periods on the dependent variables. Linear mixed effects models, matched by user ID, predicted the dependent variables, including weight change, average daily steps, and frequency of physical activity tracking, based on the binary variable time period. All analyses were adjusted for the distance to goal weight. We ran models twice, once using distance to goal weight as a random factor and another time as a fixed factor. Where a comparative analysis of variance (ANOVA) revealed a significant difference between the models, the model with the lower Akaike Information Criterion (AIC) was determined to be the best-fitting model. Where no significant difference was found in the ANOVA, the model with the lower degrees of freedom was chosen. Only the best-fitting models are reported here. To assess the sensitivity of our findings to the normality assumption in the random effects model, we compared the outputs with equivalent models fitted using generalized estimating equation (GEE; results presented in Multimedia Appendix 1).

Our first hypothesis was that motivation to lose weight and track activity would remain high, but frustrating weight measurements would drive the user to stop weight tracking. Consequently, it follows that there would be no association between the binary time period variable and average daily steps and physical activity tracking frequency. However, time period would significantly predict weight change, as weight loss would be expected to lessen in the pre-stop period.

Our second hypothesis stated that users would lose motivation for weight loss behaviors, and the resulting unsatisfactory weight feedback would lead to a stop in weight tracking. Consequently, it follows that the binary time period variable would significantly predict physical activity levels, such that there would be a decline in average daily steps from the continuous to pre-stop periods. Time period would also significantly predict weight change, as users would be expected to have less satisfactory weight measurements after reducing their weight loss efforts in the pre-stop period. There were no specific predictions about the pattern of physical activity tracking frequency in this hypothesis.

**Figure 2:**
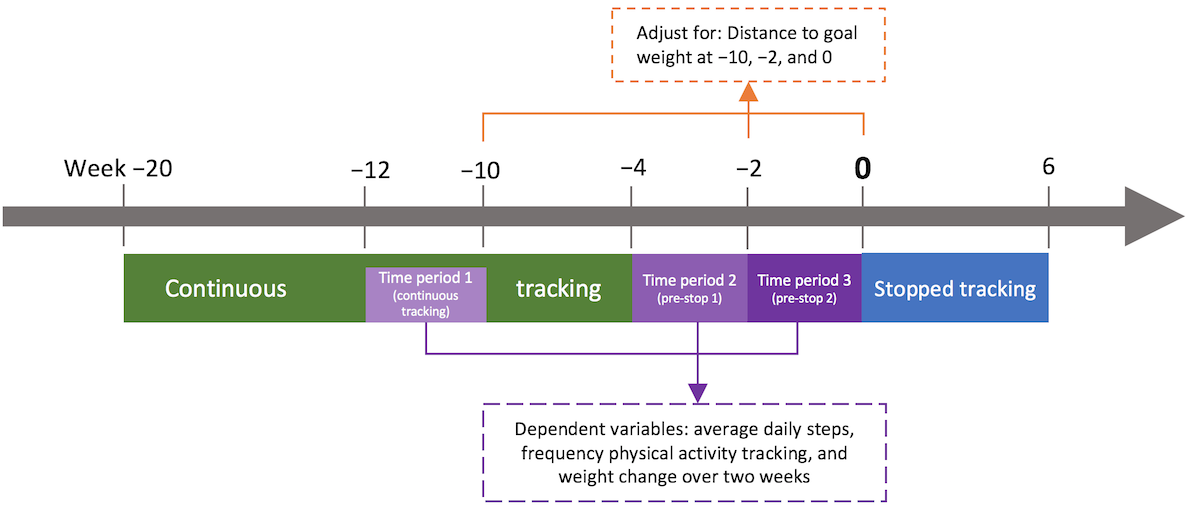
Design of the post hoc analysis, aiming to investigate temporal sequence of effects.

To identify any temporal sequences of the abovementioned hypothesized effects, we ran post hoc analyses splitting the pre-stop period (weeks −4 to 0) into two 2-week periods (−4 to −2 and −2 to 0). We reran the analysis mentioned above, this time comparing three time periods with each other, namely, weeks −12 to −10 (period 1: continuous tracking) as a baseline, weeks −4 to −2 (period 2: pre-stop 1), and weeks −2 to 0 (period 3: pre-stop 2), each 15 days long. Time period was entered as a factor in the analysis. All analyses were run twice and adjusted for the distance to goal weight, once set as a random factor and once as a fixed factor. We report the better fitting models. Tukey-adjusted post hoc comparisons investigated pairwise comparisons of the three time periods. Again, the GEE models were run to assess the sensitivity of our findings to the normality assumption (results presented in Multimedia Appendix 1). The design of this analysis is depicted in Figure 2.

#### Research Question 2

**Figure 3:**
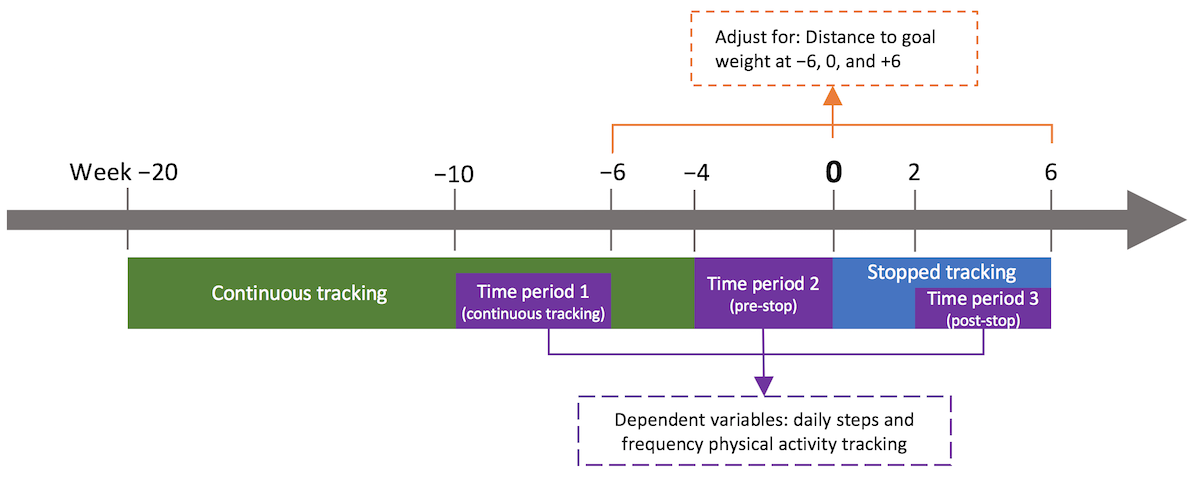
Case-crossover study design to establish linear mixed effects models for research question 2.

For our second RQ, we ran analyses to compare daily steps and physical activity tracking frequency before and after the stop in weight tracking. Time period 1 (continuous tracking) for this analysis stemmed from weeks −10 to −6 of the continuous tracking phase, which ensured that users had at least three physical activity measurements per week. This time period was treated as the baseline. Time period 2 (pre-stop) comprised the 4 weeks preceding the stop in weight tracking (weeks −4 to 0). Period 3 (post-stop) comprised weeks 2 to 6 of the break in weight tracking (see Figure 3). The analyses were run separately for daily steps and frequency of physical activity tracking using linear mixed effects models. The analyses were adjusted for the distance to goal weight at the end of the three analysis time periods. Tukey-adjusted post hoc comparisons investigated pairwise comparisons of the three time periods.

Descriptive statistics explored unadjusted differences in daily steps between the three time periods. Linear mixed effects models predicted the dependent variable daily steps from the variables time period and date of measurement. A sequential testing approach was used:

1. Random effect: user ID; fixed effect: time period
2. Random effect: user ID; fixed effects: time period, date of measurement, and interaction time period×date of measurement
3. Random effect: user ID; fixed effects: time period, date of measurement, and interaction time period×date of measurement; adjusting for distance to goal weight.

The generalized variance inflation factor (GVIF^(1/[2×df])^) was calculated at stage 2 of sequential testing to check for multicollinearity of the predictors time period and date of measurement. The third model was run twice, once entering distance to goal weight as a fixed factor and another time as a random factor. In this paper, we only present the results of the best-fitting model, which again was identified through an ANOVA and AIC comparison. We also ran GEE on the best-fitting model to test sensitivity to the normality assumption (results presented in Multimedia Appendix 1).

The hypothesis predicted a significant main effect for time period, such that daily steps would significantly decrease across all three time periods. We also expected a significant interaction term for time period and date of measurement, as the date of measurement should only be a significant negative predictor in the second and third time periods. These findings would support the hypothesis that ceasing weight tracking leads to a decline in weight loss efforts.

The frequency of physical activity tracking was computed for the same three time periods, that is, weeks −10 to −6 (continuous tracking), −4 to 0 (pre-stop), and +2 to +6 (post-stop, 29 days each). Descriptive statistics explored unadjusted differences in physical activity tracking frequency between the three time periods. A linear mixed effects model predicted physical activity tracking frequency, with user ID as a random effect and time period as a fixed effect. The analysis was adjusted for the distance to goal weight at the end of each time period. Again, distance to goal weight was entered once as a random factor and once as fixed factor, and only the best-fitting model is reported.

For our hypothesis to be correct, we expected to find significant decreases in tracking frequency across the three time periods, as this would indicate that the stop in weight tracking signals a stop in wanting to monitor weight loss efforts.

## Results

### Sample

**Figure 4:**
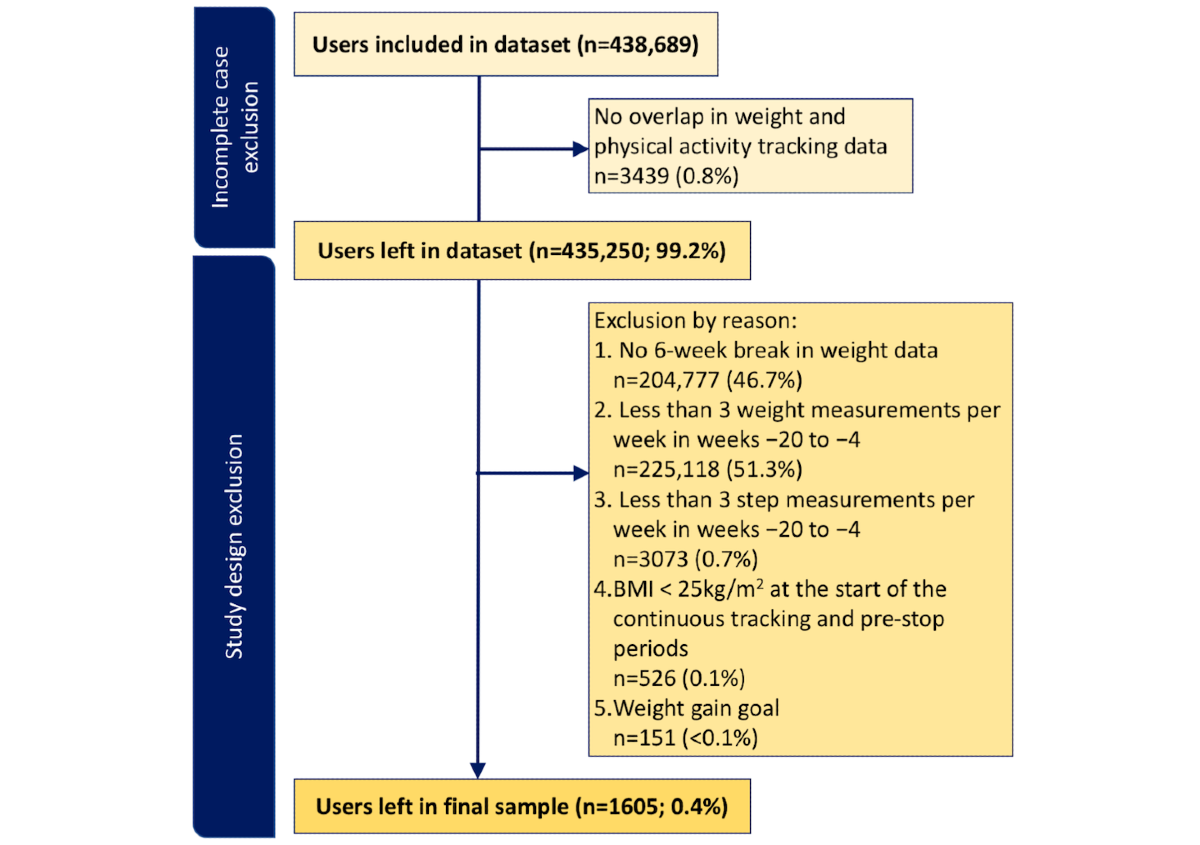
Flow diagram of the exclusion rates at each step.

The final sample consisted of 1318 male and 287 female users, the average age was 49.0 years (SD 12.5), and the average BMI at week −14 was 30.2 kg/m^2^ (SD 4.7). The reasons for exclusion are shown in Figure 4. The final dataset covered 221,173 weight and 113,162 physical activity measurements. Nearly half (778/1605, 48.47%) of the users included in the analysis were based in Europe, 36.39% (584/1605) in North America, and the rest split across other continents.

### Research Question 1

#### Weight Change

**Figure 5:**
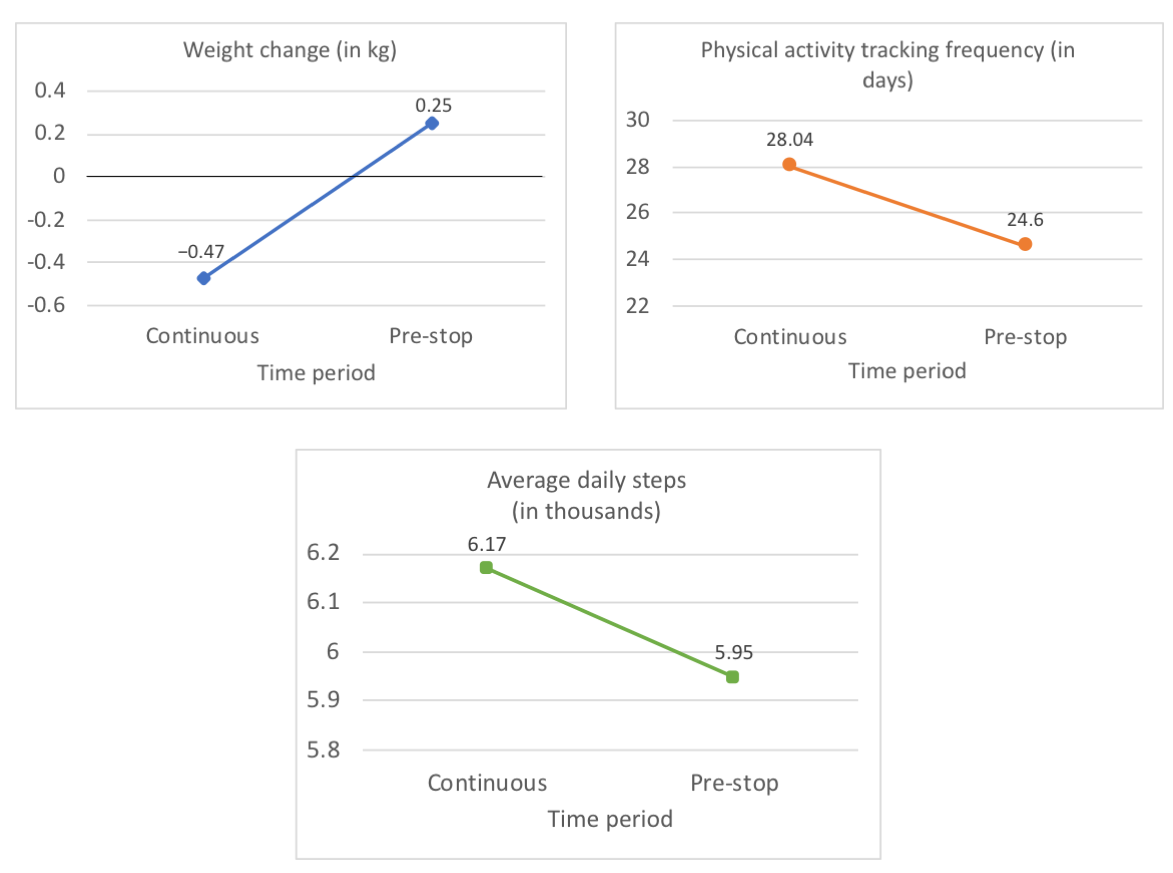
Results of the three linear mixed effects models for research question 1.

During the continuous tracking period, participants lost weight (mean −0.47 kg, SD 1.73), but pre-stop, they gained weight (mean 0.25 kg, SD 1.62). In the mixed effects model, the time period significantly predicted weight change, revealing a 0.71 kg (95% CI 0.60 to 0.81) mean difference in weight change from the continuous to pre-stop period (see Figure 5).

#### Physical Activity Tracking Frequency

Participants recorded physical activity on 3.44 (95% CI −3.78 to −3.10) fewer days during the pre-stop period compared with the continuous tracking period (see Figure 5).

#### Average Daily Steps

A total of 19 users completely stopped tracking their physical activity during the pre-stop period, meaning that average daily steps could not be calculated. These users were excluded, leaving 1586 in the analysis. Pre-stop, participants took 220 (95% CI −320 to −210) fewer steps per day than during continuous tracking (see Figure 5).

#### Post Hoc Analyses

**Figure 6:**
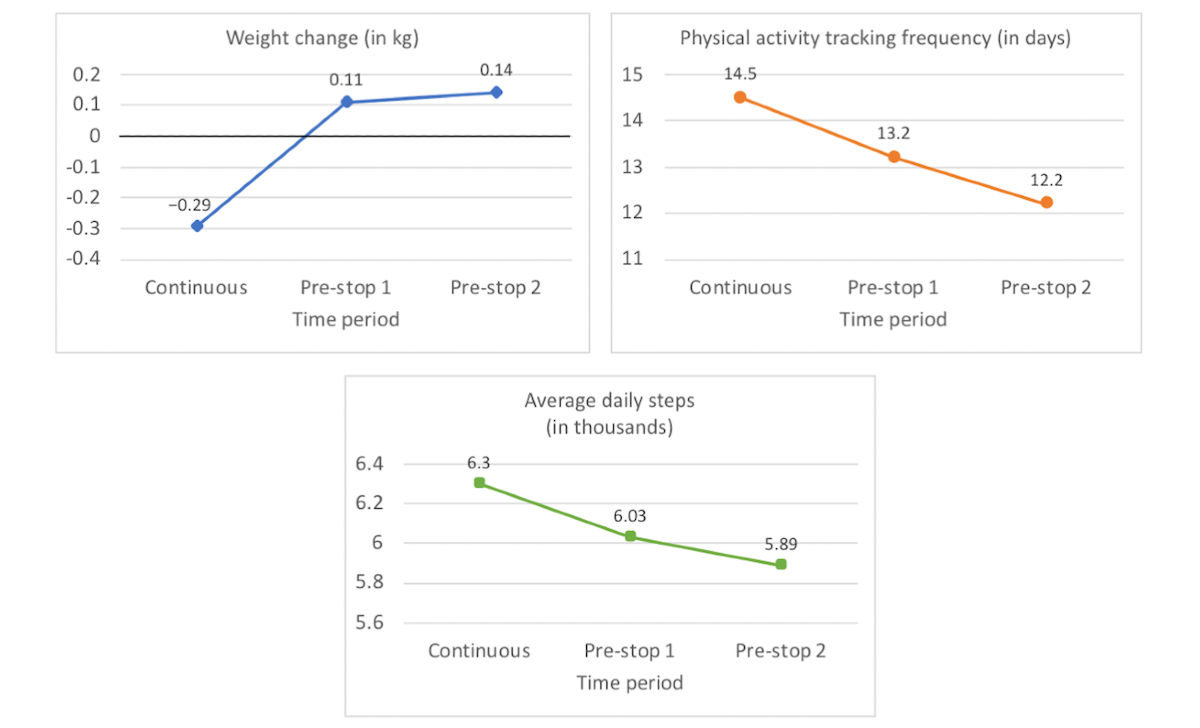
Results of the three linear mixed effects models of the post hoc analysis.

As in the prespecified analysis, users lost weight during the 2 weeks of continuous tracking (mean −0.29, SD 1.23) and gained weight in the first and second half of the pre-stop period (pre-stop 1: mean 0.11, SD 1.28; pre-stop 2: mean 0.14, SD 1.28). Post hoc comparisons revealed a 0.40 kg (95% CI 0.31 to 0.49) and 0.43 kg (95% CI 0.34 to 0.51) mean increase in weight change between the continuous tracking period and the two time periods preceding the stop in weight tracking, respectively. The two pre-stop periods did not differ significantly from each other (mean increase of 0.03 kg; 95% CI −0.06 to 0.11). The results of the best-fitting model are presented in Table 1 and Figure 6.

Physical activity tracking frequency significantly decreased by 1.28 days (95% CI −1.48 to −1.09) between the continuous tracking period and pre-stop 1. It further decreased significantly by 0.96 days (95% CI −1.16 to −0.77) between the pre-stops 1 and 2. The results of the best-fitting model are presented in Table 1 and Figure 6.

In total, 126 users completely stopped tracking their physical activity during the two pre-stop periods, meaning that average daily steps could not be calculated. We excluded these users from the analysis, reducing the sample size to 1479 users. Post hoc comparisons revealed that physical activity significantly decreased by an average of 180 steps (95% CI −290 to −70) between the continuous tracking period and pre-stop 1 and another 130 steps (95% CI −240 to −20) between the first and second half of the pre-stop period. The results of the best-fitting model are presented in Table 1 and Figure 6.

**Table 1:** Best-fitting models for the three dependent variables in the post hoc analyses.

Best-fitting model		Dependent variables					
		Weight change (kg)^a^		Physical activity tracking frequency (days)^b^		Average steps (thousands)^c^	
**Coefficients, beta (95% CI)^d^**							
	Continuous vs pre-stop 1		.40 (0.31 to 0.49)		−1.28 (−1.48 to −1.09)		−.18 (−0.29 to −0.07)
	Continuous vs pre-stop 2		.43 (0.34 to 0.51)		−2.25 (−2.44 to −2.05)		−.31 (−0.42 to −0.20)
**Tukey-adjusted post hoc comparisons, beta (95% CI)^d^**							
	Continuous vs pre-stop 1		.40 (0.31 to 0.49)		−1.29 (−1.48 to −1.09)		−.18 (−0.29 to −0.07)
	Continuous vs pre-stop 2		.43 (0.34 to 0.52)		−2.25 (−2.44 to −2.05)		−.31 (−0.42 to −0.20)
	Pre-stop 1 vs pre-stop 2		.03 (−0.06 to 0.11)		−.96 (−1.16 to −0.77)		−.13 (−0.24 to −0.02)

^a^Fixed effects: time period; random effects: distance to goal weight and user ID.

^b^Fixed effects: time period and distance to goal weight; random effects: user ID.

^c^Fixed effects: time period and distance to goal weight; random effects: user ID.

^d^Coefficients represent mean differences between the three time periods.

### Research Question 2

#### Daily Steps

Post hoc comparisons of the first mixed effects model, entering time period as a fixed factor and user ID as a random factor, showed a significant decrease of 200 daily steps (95% CI −250 to −150) from the continuous tracking to pre-stop period and a significant increase of 120 daily steps (95% CI 60 to 180) from the pre-stop to post-stop period.

**Figure 7:**
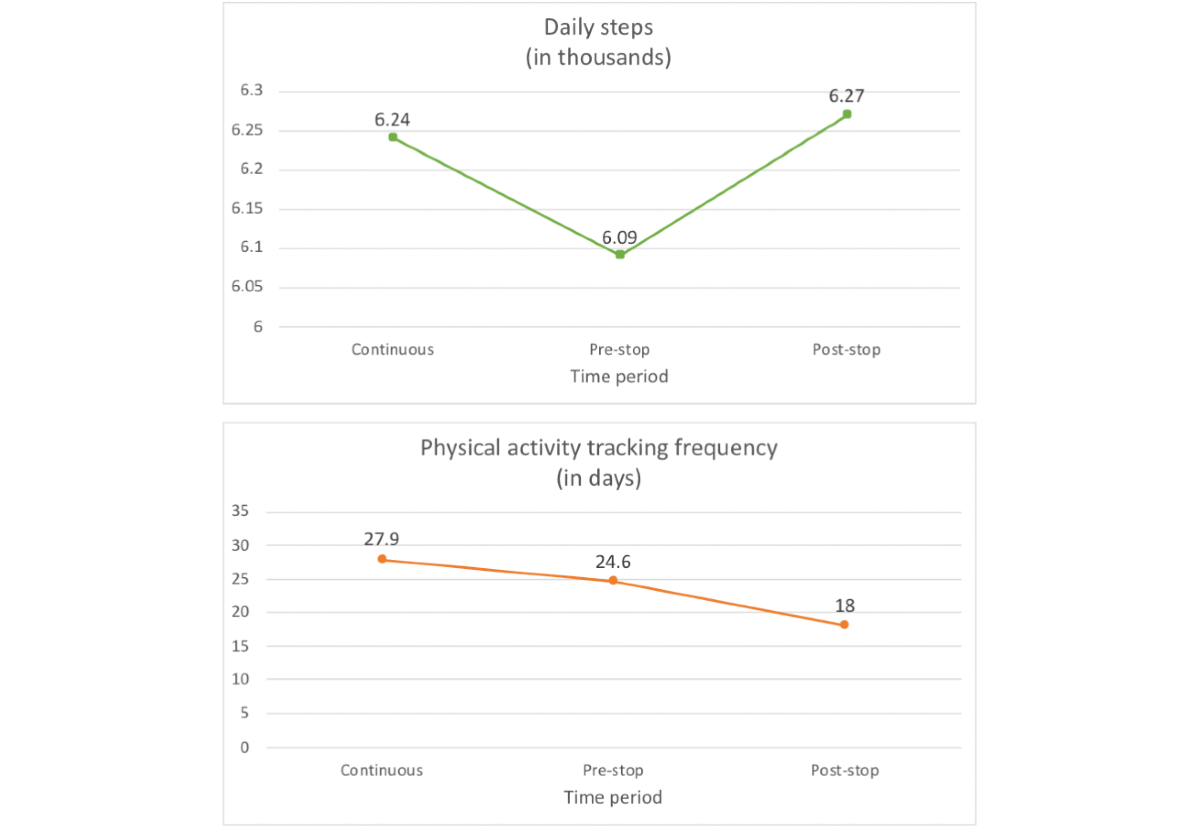
Results of the linear mixed effects models for research question 2.

In a second model, we added the variable date of measurement and the interaction term time period×date of measurement to see whether there were within-period effects, but the GVIF^(1/[2×df])^ values for the time period variable and the interaction term were above 50, indicating strong multicollinearity. We, therefore, excluded the date of measurement variable from all further analyses. In the final stage of sequential testing, post hoc comparisons of the mixed effects model adjusting for the distance to goal weight revealed a significant decrease of 190 steps (95% CI −240 to −130) from the continuous to pre-stop period. It also revealed a significant increase of 110 steps (95% CI 50 to 170) from the pre-stop to post-stop period. A small but significant decrease of 70 steps (95% CI −130 to −10) was found in the comparison between the continuous tracking and the post-stop period (see Figure 7). A summary of all results can be found in Table 2.

**Table 2:** Results of the daily steps data analysis of research question 2.

Daily steps data analysis		Model 1^a^	Model 2^b^	Model 3^c^
**Coefficients, beta (95% CI)^d^**				
	Continuous vs pre-stop	−.20 (−0.25 to −0.15)	−2.12 (−4.79 to 0.54)	−.19 (−0.24 to −0.13)
	Continuous vs post-stop	−.08 (−0.14 to −0.02)	−6.16 (−9.19 to −3.12)	−.07 (−0.13 to −0.01)
	Date of measurement	N/A^e^	−.00 (−0.00 to 0.00)	N/A
	Continuous vs pre-stop×date of measurement	N/A	−.00 (−0.00 to 0.00)	N/A
	Continuous vs post-stop×date of measurement	N/A	−.00 (−0.00 to 0.00)	N/A
**Tukey-adjusted post hoc comparisons, beta (95% CI)^d^**				
	Continuous vs pre-stop	−.20 (−0.25 to −0.15)	N/A	−.18 (−0.24 to −0.13)
	Continuous vs post-stop	−.08 (−0.14 to −0.02)	N/A	−.07 (−0.13 to −0.01)
	Pre-stop vs post-stop	.12 (0.06 to 0.18)	N/A	.11 (0.05 to 0.17)

^a^Fixed effects: time period; random effects: user ID.

^b^Fixed effects: time period, date of measurement, and interaction term time period×date of measurement; random effects: user ID.

^c^Fixed effects: time period; random effects: distance to goal weight and user ID.

^d^Coefficients represent mean differences between the three time periods.

^e^Not applicable.

#### Physical Activity Tracking Frequency

Post hoc comparisons revealed that physical activity tracking frequency significantly decreased by 3.3 days (95% CI −3.82 to −2.85) between the continuous tracking and pre-stop period and another 6.6 days (95% CI −7.12 to −6.16) between the pre-stop and post-stop period. The results of the best-fitting model are presented in Table 3 and Figure 7.

**Table 3:** Results of the physical activity tracking frequency analysis of research question 2.

Physical activity tracking frequency analysis		Model^a^
**Coefficients, beta (95% CI)^b^**		
	Continuous vs pre-stop	−3.33 (−3.82 to −2.85)
	Continuous vs post-stop	−9.98 (−10.50 to −9.50)
**Tukey-adjusted post hoc comparisons, beta (95% CI)^b^**		
	Continuous vs pre-stop	−3.34 (−3.82 to −2.86)
	Continuous vs post-stop	−9.98 (−10.50 to −9.50)
	Pre-stop vs post-stop	−6.64 (−7.12 to −6.16)

^a^Fixed effects: time period and distance to goal weight; random effects: user ID.

^b^Coefficients represent mean differences between the three time periods.

## Discussion

### Principal Findings

The analyses targeting the first RQ revealed that a stop in weight tracking is preceded by decreased step counts, lower physical activity tracking frequencies, and weight gain. The findings thus counter our first hypothesis, which had stated that physical activity (ie, motivation to lose weight) would remain stable, whereas a weight gain would precede the stop in weight tracking. The post hoc analysis showed that the changes in weight and physical activity developed concurrently, as effects appeared at the same time, in pre-stop 1. The results, therefore, only partially support our second hypothesis, as they do not reveal the sequential effect theorized: first, a decrease in physical activity, followed by weight gain and a stop in weight tracking.

Regarding the second RQ, we found a decrease in the frequency of physical activity monitoring but an increase in physical activity on days when activity was recorded after users stopped tracking their weight. Our hypothesis, which stated that users would show a decrease in both physical activity levels and tracking frequency, is, therefore, only partially supported.

### Users Gain Weight Before They Stop Tracking Their Weight

The literature provides abundant cross-sectional evidence that ceasing regular weighing and weight gain are associated [6-8,29,30]. This has been interpreted to indicate that reduced tracking frequency leads to weight gain. However, our findings suggest that the relationship could be reversed: in this analysis, weight gain preceded the stop in weight monitoring. We reached a similar conclusion in our recent review of the qualitative literature of experiences of self-directed weight loss [31]. These results can be explained in terms of *The Ostrich Problem*, which proposes that people avoid outcome information when it shows that progress is poor or it elicits negative emotions [32,33]. In line with this, previous research on weight loss has shown that people who anticipate negative feedback from the scales choose not to weigh themselves to avoid negative feelings [17,34-36]. In a study by Mintz et al [21], 63.1% (99/157) of the female participants reported reacting emotionally to weight measurements, and half of the participants felt that the weight measurements affected their feelings of self-worth. Taken together, it seems that some people struggle receiving negative feedback from the scales and, therefore, stop exposing themselves to the information. The lack of constructive use of weighing feedback, hence, leads to a stop in engagement with self-monitoring, which is a necessary step for self-regulation. The insights provided by these findings open up avenues for intervention because it is plausible that helping users reframe negative weight measurements as constructive feedback could aid successful self-regulatory processes.

### Users Reduce Weight Loss Efforts Before Ceasing Weight Tracking

Users engaged in less physical activity and reduced the frequency of monitoring physical activity before they stopped tracking their weight. As attempting to increase energy expenditure through physical activity is one of the most common approaches to weight loss [21-23], we interpret these changes as reductions in the motivation to lose weight, leading users to stop weight tracking. Previous research also supports the notion that levels of motivation might be connected to weight monitoring adherence. One study reported that autonomous motivation predicted adherence to self-monitoring [37]. Two further studies found that measures related to motivation and weight loss behaviors, including goal ownership, weight loss expectations, and estimated weight loss skills, were negatively associated with subsequent dropout from weight loss treatment [38,39].

The decline of physical activity and increase in weight occurred concurrently, and it is possible that the two aspects may have influenced and reinforced each other. That is, reduced motivation to lose weight and thus reduced physical activity might have led to weight gain, which, in turn, might have reinforced the decline in motivation to track weight, bringing the whole self-regulatory system to a halt. There is a little empirical evidence to support this, including a study by Webber et al [37] who found that the positive association between autonomous motivation and adherence to self-monitoring was mediated by weight loss.

### Consequences of Stopping Weight Monitoring

The question arises why users reduced the frequency of physical activity monitoring but increased their daily step count after stopping weight monitoring. There is some evidence that users are more likely to track favorable weight measurements [25], and it is thus conceivable that users tended to only wear their tracking device on their more active days, leading to less tracking but higher average physical activity on days with data. In addition, users may have had less interest in monitoring their physical activity, given that they could no longer put the measurements into context with weight data, a prerequisite to evaluating behavior and progress for self-regulation. In line with this hypothesis, qualitative studies of people who autonomously track health parameters have reported that a common aim is to identify correlations between the measurements of their different health parameters [36,40] to gain a better understanding of their body and to experiment with different ideas, for example, whether more sleep helps with weight loss [36]. Tracking more than one parameter has been associated with better adherence overall, possibly because of mutual reinforcement [41]. Having no longer collected information about their weight and thus being no longer able to complete the self-regulation process, the users in our sample might have perceived less value in their physical activity data, leading to a reduced tracking frequency.

It is notable that although the frequency of physical activity monitoring declined, users did not stop monitoring their physical activity completely, although they had stopped monitoring their weight. One reason for this discrepancy might be that daily physical activity measurements are independent of each other, whereas weight measurements are highly autocorrelated. That is, although a weight measurement from one day necessarily predicts the weight measurement of the next day, physical activity measurements are reset to zero at the start of each day, and the participant might, therefore, find it easier to start afresh.

### Strengths and Limitations

One strength of this analysis is that it examines the patterns of self-monitoring behavior in a setting in which the users were not aware that they were being observed. This reduces biases such as the Hawthorne effect [37,42], increasing the external validity of our findings. Our sample size was considerably larger than most researcher-led studies. Covering 221,173 weight and 113,162 physical activity measurements from 1605 users, our analysis was well powered to detect pattern changes in the data. Context information for the users was, however, sparse. The dataset includes a high proportion of men, which is rare in the context of weight loss studies, but we lack information regarding socioeconomic status and ethnicity, making it difficult to gauge generalizability. However, the spread of users across countries means it is likely to encompass a broader mix of ethnic backgrounds and lifestyles than most single-country studies. Unfortunately, we do not know whether the users we analyzed participated in any kind of weight loss program. However, as our analyses were conducted within individuals, confounding differences between individuals were removed. Another disadvantage of using context-restricted app data is that we had no information on users’ intentions. For instance, we do not know whether the stop in weight data actually reflects a stop in weight tracking. Users might instead have switched to a different app to track their weight measurements. We designed the study to minimize this possibility. The minimum period of continuous tracking of 16 weeks reduced the chance that the users were just *trying out* the app [43]. Only one-fourth of the downloaded health apps are used long term, and the cutoff lies around the tenth use [44]. Users of tracking apps become increasingly bound to their app of choice, as data on past physical activity and weight measurements cannot easily be transferred between apps [11]. We can, therefore, assume that after 16 weeks of continuous tracking, users who stop tracking their weight did not simply switch apps. It, however, remains possible that users continued weighing themselves without synchronizing the data to the app. Nevertheless, because we observed a significant weight gain and decrease in physical activity before the stop in weight tracking, it seems that the stop in weight tracking did occur in the context of waning weight loss efforts. Unfortunately, we did not have access to matched dietary data, which would have added another important proxy measure of the motivation to lose weight to the model. We do, however, believe that physical activity should be a good indicator of motivation on its own because roughly 80% of people report increasing their physical activity as part of their weight loss efforts [21-23].

A necessary feature of our study design is that we restricted our analysis to a group of people who monitored themselves very frequently. Individuals who self-monitor very frequently are likely to represent a highly motivated population [7], but this limitation is comparable with that of any other weight loss study where people who choose to take part have an intrinsic motivation to make a weight loss attempt. A final limitation is that because the design of this research is observational, we are unable to make causal inferences from the data. Future research using other designs is necessary to further investigate the patterns we identified.

### Conclusions

This observational analysis shows that before people who weighed themselves regularly cease doing so, their weight increases and their physical activity intensity and tracking frequency declines. This probably indicates that a stop in regular weighing occurs in the context of a decline in weight loss efforts. After ceasing to track weight, there is a decline in the frequency of monitoring physical activity and an increase in the daily steps taken on the days monitored. The stop in weighing can be interpreted as a halt in self-regulation for weight loss, as progress is no longer monitored and cannot be evaluated.

Our results indicate that phases of concurrent weight gain and decreases in physical activity constitute an appropriate time for intervention. Programs tackling declining motivation and helping with the constructive use of weight monitoring may, therefore, have the potential to help users stay on track with self-regulation and their weight loss efforts.

## Appendix

Multimedia Appendix 1 Results of GEE Models.

## Abbreviations

AIC: Akaike Information Criterion
ANOVA: analysis of variance
CLAHRC: Collaboration for Leadership in Applied Health Research and Care Oxford
GEE: generalized estimating equation
GVIF: generalized variance inflation factor
NHS: National Health Service
NIHR: National Institute for Health Research
RQ: research question
