# Supplementary material for: Patterns in Weight and Physical Activity Tracking Data Preceding a Stop in Weight Monitoring: Observational Analysis
Source: J Med Internet Res. 2020 Mar 17;22(3):e15790. doi: 10.2196/15790 (PMC7109615; doi:10.2196/15790)
Supplement: Multimedia Appendix 1 [file jmir_v22i3e15790_app1.pdf]

Results of GEE Models

Research Question 1

Table A. Results of the GEE models testing sensitivity to the normality assumption for pre-specified analyses of RQ1.

Variable	β	*P*
Weight Change	0.73	<.001
Physical Activity Tracking Frequency	-3.4	<.001
Average Daily Steps	-0.22	.059

Table B. Results of the GEE models testing sensitivity to the normality assumption for post-hoc analyses of RQ1.

Variable		β	*P*
Weight Change			
	TP1 to TP2	0.40	<.001
	TP1 to TP3	0.43	<.001
	TP2 to TP3	0.03	.85
Physical Activity Tracking Frequency			
	TP1 to TP2	-1.3	<.001
	TP1 to TP3	-2.3	<.001
	TP2 to TP3	-1.0	<.001
Average Daily Steps			
	TP1 to TP2	-0.18	.007
	TP1 to TP3	-0.31	<.001
	TP2 to TP3	-0.13	.014

Research Question 2

Table C. Results of the GEE models testing sensitivity to the normality assumption for pre-specified analyses of RQ2.

Variable		β	*P*
Physical Activity Tracking Frequency			
	TP1 to TP2	-3.3	<.001
	TP1 to TP3	-10.0	<.001
	TP2 to TP3	-6.6	<.001
Average Daily Steps			
	TP1 to TP2	-0.14	.011
	TP1 to TP3	0.04	.879
	TP2 to TP3	0.18	.060
